# The Multi-Drug Resistant Tuberculosis Diagnosis and Treatment Cascade in Bangladesh

**DOI:** 10.1371/journal.pone.0129155

**Published:** 2015-06-25

**Authors:** Sarder Tanzir Hossain, Petros Isaakidis, Karuna D. Sagili, Shayla Islam, Md Akramul Islam, Hemant Deepak Shewade, S. M. Mostofa Kamal, Ashaque Husain

**Affiliations:** 1 TB Control Programme, BRAC, Dhaka, Bangladesh; 2 MédecinsSansFrontières, Operational Research Unit, Luxembourg City, Luxembourg; 3 International Union Against Tuberculosis and Lung Disease (The Union), South-East Asia Regional Office, New Delhi, India; 4 Mahatma Gandhi Institute of Medical Sciences (MGIMS), Sevagram, Wardha, India; 5 NationalTuberculosis Reference Laboratory, NIDCH, Dhaka, Bangladesh; 6 National Tuberculosis Control Programme, DGHS, Dhaka, Bangladesh; Public Health Research Institute at RBHS, UNITED STATES

## Abstract

**Objectives:**

To determine, in areas supported by BRAC, Bangladesh i) the pre-diagnosis and pre-treatment attrition among presumptive and confirmed Multi-Drug Resistant Tuberculosis (MDR-TB) patients and ii) factors associated with attrition.

**Methods:**

This was a retrospective cohort study involving record review. Presumptive MDR-TB patients from peripheral microscopy centres serving 60% of the total population of Bangladesh were included in the study. Attrition and turnaround time for MDR-TB diagnosis by Xpert MTB/RIF and treatment initiation were calculated between July 2012 and June 2014.

**Results:**

Of 836 presumptive MDR-TB patients referred from 398 peripheral microscopy centres, 161 MDR-TB patients were diagnosed. The number of diagnosed MDR-TB patients was less than country estimates of MDR-TB patients (2000 cases) during the study period. Among those referred, pre-diagnosis and pre-treatment attrition was 17% and 21% respectively. Median turnaround time for MDR-TB testing, result receipt and treatment initiation was four, zero and five days respectively. Farmers (RR=2.3, p=0.01) and daily wage laborers (RR=2.1, p=0.04) had twice the risk of having pre-diagnosis attrition. Poor record-keeping and unreliable upkeep of presumptive MDR-TB patient databases were identified as challenges at the peripheral microscopy centres.

**Conclusion:**

There was a low proportion of pre-diagnosis and pre-treatment attrition in patients with presumptive and confirmed MDR-TB under programmatic conditions. However, the recording and reporting system did not detect all presumptive MDR-TB patients, highlighting the need to improve the system in order to prevent morbidity, mortality and transmission of MDR-TB in the community.

## Introduction

Despite progress in the detection of Multi-Drug Resistant/Rifampicin Resistant Tuberculosis (MDR/RR-TB) cases, major diagnostic gaps remain: 55% of reported TB patients estimated to have MDR-TB were not detected in 2013 [1].

Timely identification of MDR-TB cases and prompt initiation of treatment are crucial to prevent the transmission of disease and reduce related high morbidity and mortality [2]. Studies worldwide in the past have documented pre-diagnostic (21%-90%) and pre-treatment attrition in the pathway of presumptive MDR-TB patients [3–9]. Though introduction of molecular diagnostic techniques has resulted in a decrease in laboratory Turn Around Time (TAT), operational issues still remain a major concern.

Bangladesh is one of the 27 high burden countries for MDR-TB [1]. The National TB Programme (NTP) of Bangladesh has been implementing Programmatic Management of Drug Resistant TB (PMDT) since August 2008 in order to control the epidemic. In 2013, there were 2100 (1.4%) and 2600 (29%) estimated MDR-TB cases among the notified pulmonary new and re-treatment TB cases respectively.

According to Bangladesh NTP policy, all presumptive MDR-TB cases should be tested by Xpert MTB/ RIF. However, out of an estimated 4700 MDR-TB patients in 2013, NTP Bangladesh diagnosed only 1024 MDR/RR TB cases and only 684 MDR–TB patients enrolled for treatment in 2013 [1]. Therefore, there are concerns that not all presumptive MDR-TB cases are getting tested (pre-diagnosis attrition) resulting from considerable delays between referral and actual testing. Similarly, there are concerns that important delays occur between the diagnosis of MDR-TB and its treatment initiation (pre-treatment attrition). To date, such information remains anecdotal and there has been no systematic study to provide evidence that could guide interventions to prevent pre-diagnosis and pre-treatment attrition in the Bangladesh NTP. BRAC, an international development non-governmental organization, present in more than 10 countries around the world, is currently the largest partner of the Bangladesh NTP.

This operational research was conducted i) to determine the pre-diagnosis and pre-treatment attrition among presumptive and confirmed MDR/RR-TB patients and ii) to study factors associated with attrition, in areas supported by BRAC in Bangladesh, between July 2012 and June 2014.

## Methods

### Ethics

Ethics approval was obtained from the Bangladesh Medical Research Council (BMRC), Dhaka and the Ethics Advisory Group, International Union Against Tuberculosis and Lung Disease, Paris, France. As this study involved collection of routine programme data, informed consent from the patients was waived by the ethics committee.

### Study design

This was a retrospective cohort study involving record review of presumptive MDR-TB patients.

### Setting

#### General setting

Bangladesh is situated in the north-eastern part of South Asia. It has a population of over 150 million and administratively it is divided in seven divisions, 64 districts, 488 sub-districts (upazilas) and 4,466 unions [10]. Bangladesh is unique in that it has one of the highest population densities in the world; it is one of the high burden countries for TB; and has a low prevalence of HIV [11]. The BRAC-TB Control Programme started in 1984 as a pilot project in Manikganj Sadar upazila and now covers 297 upazilas in 42 districts with a population of 93 million people, including the remote mountain areas of Chittagong Hills [12].

#### Study setting

In collaboration with the NTP, this study was carried out in 42 districts and one city corporation area of Sylhet covered by BRAC. BRAC supports a population of 93 million which is approximately 60% of the Bangladeshi population. These areas contain 398 microscopy laboratories, 18 Xpert MTB/RIF laboratories including the Regional TB Reference Laboratory (RTRL), the National TB Reference Laboratory (NTRL), and Chest Diseases Hospitals and National Institute of Diseases of the Chest and Hospital (NIDCH) of Dhaka plus some other specialized hospitals.

The criteria used to identify presumptive MDR-TB in the NTP are (any one): i) new case—treatment failure, ii) retreatment case—treatment failure, iii) symptomatic contacts of confirmed MDR-TB cases, iv) new cases who didn’t convert smear by three months, v) Category II patients who did not convert smear by four months, vi) any treatment relapse, vii) any return after default, viii) all TB-HIV co-infected patients at the start of TB treatment, and ix) others (unknown treatment history or any smear negative or extra-pulmonary TB patient clinically not improved in spite of treatment as per NTP guidelines).

All presumptive MDR-TB patients from peripheral microscopy laboratories, except some areas where piloting of sample referral is going on, are referred to nearest Xpert MTB/RIF laboratory for MDR-TB testing. If diagnosed as MDR-TB, then patients are counseled to get admitted at the nearest MDR-TB treatment facilities. MDR-TB treatment is initiated with the detection of MDR/RR-TB as per the national algorithm. BRAC also provides social support in terms of financial support throughout the MDR-TB diagnosis and treatment cascade in Bangladesh.

### Patient population

All presumptive MDR-TB patients, who were referred from BRAC’s peripheral microscopy centres to Xpert MTB/RIF laboratories under areas supported by BRAC between July 2012 and June 2014, were included in the study.

### Operational definitions and data collection

The following operational definitions were used in the study: pre-diagnosis attrition applies to any presumptive MDR-TB patient who was not tested within 30 days from the date when the patient was declared “presumptive MDR-TB” based on microscopy laboratory test; and pre-treatment attrition applies to any confirmed MDR-TB patient who was not enrolled for treatment within 30 days from the date of diagnosis based on the Xpert MTB/RIF result.

A list of presumptive DR-TB patients, who were referred, was prepared from the presumptive MDR-TB register at peripheral microscopy centres. Data for this cohort were collected through a structured proforma from presumptive DR-TB registers, TB treatment cards, Xpert MTB/RIF register and MDR-TB treatment cards. Data were validated from different sources wherever possible. Data variables included age, sex, occupation, TB disease type, TB registration number, criterion for presumptive MDR-TB, name of the peripheral microscopy lab, name of the reference or Xpert MTB/RIF laboratory and DR-TB registration number. We also collected the following: date of declaration as presumptive MDR-TB, date of MDR-TB test result if done, and date of registration for MDR-TB treatment, if it was initiated.

### Analysis and statistics

We used EpiData software for data entry and analysis (version 3.1 for entry and version 2.2.2.182 for analysis, EpiData Association, Odense, Denmark). Descriptive statistics (mean, median and proportions) were used to describe the data. Relative risks (RR) and Chi- square tests were used to determine factors associated with attrition. A P value <0.05 was considered statistically significant.

## Results

**Fig 1:**
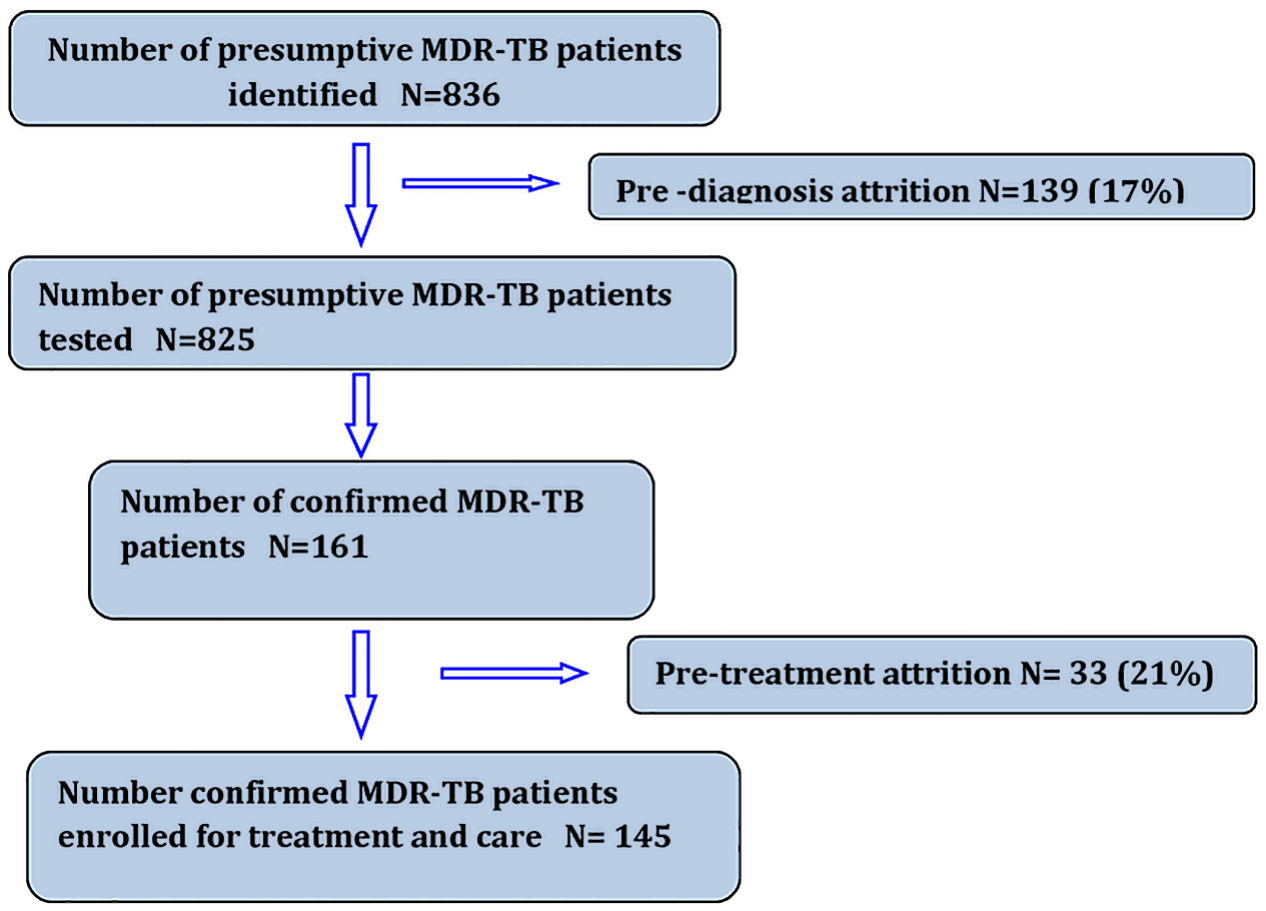
Flow chart showing pre-diagnosis and pre-treatment attrition in MDR-TB diagnosis and treatment cascade in BRAC supported regions of Bangladesh, July 2012- June 2014.

A total of 836 presumptive MDR-TB patients were identified and included in the analysis. The diagnosis and treatment cascade is shown in Fig 1. Of 836 presumptive MDR-TB patients, 575 (68.8%) were male, mean (SD) age was 41 (18.3) years and 216 (25.8%) were farmers. Half (50.4%) of the patients were referred between Jan-Jun 2014. Relapse among new and retreatment cases was the most common criterion for referral for diagnosis (38.5%) (Table 1).

**Table 1:** Baseline socio-demographic and clinical characteristics of presumptive and confirmed MDR-TB patients in BRAC supported regions of Bangladesh, July 2012- June 2014[N = 836].

	Total presumptive MDR		Total confirmed MDR	
	TB cases (N = 836)		TB cases(N = 161)	
**Examinee's sex**	**N**	**%**	**N**	**%**
Female	261	31.2	51	31.7
Male	575	68.8	110	68.3
**Age group**				
Less than 15 yr	14	1.7	5	3.1
15 to 44 yr	474	56.7	114	70.8
45 to 64 yr	248	29.7	32	19.9
More than 64 yr	100	12	10	6.2
**Occupation**				
Employed	93	11.1	15	9.3
Businessman	91	10.9	15	9.3
Farmer	216	25.8	29	18
Professionals	20	2.4	4	2.5
Not working	124	14.8	25	15.5
Daily wage labourers	242	28.9	46	28.6
Not recorded	50	6	27	16.8
**Type of TB**				
Pulmonary TB	818	97.8	161	100
Extra pulmonary TB	15	1.8	0	0
Not recorded	3	0.4	0	0
**Criteria of presumptive MDR-TB patients**				
Not recorded	6	0.7	0	0
Failure of cat I and II	151	18.1	52	32.3
Contacts of MDRTB	3	0.4	2	1.2
Non converters of cat I and II	182	21.8	35	21.7
All relapses	322	38.5	60	37.3
All return after default	34	4.1	7	4.3
TB-HIV co- infection	12	1.4	0	0
Others	126	15.1	5	3.1
	**836**	**100**	**161**	**100**

Pre-diagnosis attrition was found in 139 (16.6%) patients (Table 2). Among those who who underwent testing (n = 825), the median (IQR) TAT was four (2, 14) days. The median (IQR) time taken for Xpert MTB/RIF test results to be given to patients after testing was zero (0, 2) days.

**Table 2:** Characteristics of presumptive MDR-TB patients with pre-diagnosis attrition in BRAC supported regions of Bangladesh, July 2012- June 2014[N = 139].

	N = 139	Pre-diagnosis attrition“*”(%)	Relative risk	p- value
**Examinee's sex**				
Female	42	16.1	“**”Ref	Ref
Male	97	16.9	1.04	0.86
**Age group**				
Less than 15 yr	0	0	-	-
15 to 44 yr	76	16	Ref	Ref
45 to 64 yr	45	18.1	1.13	0.54
More than 64 yr	18	18	1.13	0.95
**Occupation**				
Employed	9	9.7	Ref	Ref
Businessman	14	15.4	1.59	0.34
Farmer	48	22.2	2.28	0.01
Professionals	3	15.0	1.55	0.76
Not working	13	10.5	1.08	0.97
Daily wage labourers	48	19.8	2.04	0.04
Not recorded	4	8.0	-	-
**Type of TB**				
Pulmonary TB	134	16.4	Ref	Ref
Extra pulmonary TB	5	33.3	2.03	0.163
Not recorded	0	0		
**Criteria of presumptive MDR-TB patients**				
Failure of cat I and II	24	15.9	1.80	0.43
Contacts of MDRTB	0	0		0
Non converters of cat I and II	30	16.5	1.88	0.37
All relapses	50	15.5	1.76	0.42
All return after default	3	8.8	Ref	Ref
TB-HIV co- infection	0	0	-	-
Others	31	24.6	2.79	0.07
Not recorded	1	16.7	-	-
	**139**	**16.6**		

“*”Row percentage

“**”Reference value to calculate relative risk

The number of MDR-TB cases diagnosed and referred for DR-TB treatment among the cohort was 161 (19.3%). Of those, 110 (68.3%) were male, the mean (SD) age was 35 (17) years, 29 (18%) were farmers and all were pulmonary cases. Pre-treatment attrition was seen in 33 (20.5%) patients (Table 3). Among those who were registered for DR-TB treatment (n = 145), median (IQR) TAT to start treatment after receiving test results was five (2, 15) days.

**Table 3:** Characteristics of presumptive MDR-TB patients with pre-treatment attrition in BRAC supported regions of Bangladesh, July 2012- June 2014[N = 33].

	N = 33	Pre-treatment attrition“*” (%)	Relative risk	p-value
**Examinee's sex**				
Female	10	19.6	“**”Ref	Ref
Male	23	20.9	1.97	0.98
**Age group**				
Less than 15 yr	1	20	2	0.79
15 to 44 yr	22	19.3	1.93	0.76
45 to 64 yr	9	28.1	2.81	0.18
More than 64 yr	1	10	Ref	Ref
**Occupation**				
Employed	4	26.7	2.23	0.45
Businessman	2	13.3	1.10	0.71
Farmer	7	24.1	2.0	0.42
Professionals	1	25.0	2.5	0.39
Not working	3	12	Ref	Ref
Daily wage labourer	11	23.9	1.99	0.37
Not recorded	5	18.5	1.54	0.79
**Type of TB**				
Pulmonary TB	33	20.5	-	-
Extra pulmonary TB	0	0	-	-
**Criteria of confirmed MDR-TB patients**				
Failure of cat I and II	8	15.4	1.07	0.63
Contacts of MDRTB	0	0	-	-
Non converters of cat I and II	7	20	1.39	0.86
All relapses	16	26.7	1.87	0.96
All return after default	1	14.3	Ref	Ref
TB-HIV co- infection	0	0	-	-
Others	1	20	1.39	0.60
	**33**	**20.5**		

“*”Row percentage

“**”Reference value to calculate relative risk

Univariate analysis of socio-demographic factors showed that farmers (RR = 2.3, p = 0.01) and daily wage laborers (RR = 2.1, p = 0.04) had twice the risk of having pre-diagnosis attrition when compared to employed patients. Extra-pulmonary TB patients also had twice the risk of having pre-diagnosis attrition when compared to pulmonary TB patients, but this difference was not statistically significant. No factors were found to be significantly associated with pre-treatment attrition.

## Discussion

In BRAC supported areas of the Bangladesh NTP, both pre-diagnostic attrition and pre-treatment attrition were low (≤ 21%) among presumptive MDR-TB patients referred from the peripheral microscopy centres. The NTP performance in referring presumptive MDR-TB patients has been improving over time from July 2012 to June 2014 and this was an encouraging finding.

In Bangladesh about 83% patients referred for Xpert MTB/RIF testing were actually tested as compared to 10% in Tanzania [3], 39% in China [4], 40% in Malawi [5], 46% in Cambodia [6], 55% in Andhra Pradesh [7], India, 74% in Delhi, India [8] and 80% in Sri Lanka [9], all settings with phenotypic diagnostic techniques. With the introduction of molecular diagnostic techniques (LPA) in India, the uptake of testing was as high as 95% in a setting in Delhi [8]. As for MDR-TB treatment initiation after the introduction of LPA in Delhi, 88% of patients referred were actually initiated on treatment, similar to our study. Reported differences worldwide could be attributed to several factors including, variation in criteria for presumptive MDR-TB, logistics and access to services, phase of PMDT implementation, timelines used to calculate TAT, and diagnostic and treatment delays in various settings.

The median turnaround times throughout the diagnosis and treatment pathway were satisfactory in Bangladesh, especially when compared to other settings: four days for Xpert MTB/RIF testing, same day for Xpert MTB/RIF results given to the patients, and five days from confirmation of MDR-TB diagnosis to treatment initiation. In the Delhi study, for example, these median TATs were nine, five and 37 days respectively [8]. Median treatment commencement time was 17 days as a result of Xpert MTB/RIF testing in South Africa [13].

### Strengths and limitations

We acknowledge some limitations in this study. The diagnosed number of MDR-TB patients was less than the country estimates of MDR-TB patients (2000 cases) during the study period. In our study, only cases referred from microscopy centres were included, whereas in other studies some of the presumptive MDR-TB cases might come directly from other sources. Also, overlapping (BRAC and non BRAC) of services is possible even in BRAC supported areas. Moreover, it is highly likely that not all presumptive MDR-TB were being identified.

During the study, a register of presumptive MDR-TB patients was not found in several peripheral microscopy laboratories and proper documentation of presumptive MDR-TB patients was not recorded, especially between June–Dec 2012 when PMDT was being implemented. Over time, this situation has improved, yet, there is further room for improvement. Poor update of the list of presumptive MDR-TB patients in peripheral microscopy laboratory centers was also common. There were several cases in which baseline data as well as dates were incompletely entered in records. It is hypothesized that referred patients who returned with Xpert MTB/RIF results were more likely entered retrospectively into the registers. Therefore, a selection bias was likely introduced during the study which may have led to an underestimation of attrition, especially pre-diagnosis attrition.

Actual eligible referrals were not identified by the investigators. Only those identified by the programme were included as the denominator in the cohort. Many referrals were not included in the denominator because of absence of baseline data. These are inherent limitations of a record review study. Health system factors, particularly relating to the recording and registration of presumptive and confirmed tuberculosis cases, were found to be important contributors to pre-treatment loss to follow-up in several studies [14]. It will be important to systematically identify factors associated with attrition and qualitative methods may provide valuable data especially on patient and provider related factors.

Despite these limitations, our study has several strengths. This was the first study in Bangladesh to identify attrition in the diagnostic and treatment pathway of PMDT services and describe the diagnosis and treatment cascade. A-major strength of this study is that data were collected from a large area of the country (representing 60% of the population) under programmatic conditions and, thus, they are likely to be representative of the PMDT activities countrywide. A standard and strict attrition criterion of 30 days was uniformly followed for each referral. This was not the case in previous studies from other settings. Some operational challenges identified are specific to the region, though are likely to be encountered in other low and middle income countries. Data were quality assured and robust and STROBE guidelines were followed [15].

### Implications for policy and practice

Mere implementation of molecular diagnostic tests won’t suffice unless operational issues related to PMDT at field level are addressed. There is a need to improve the recording and reporting of presumptive MDR-TB cases at each level. A cohort-wise analysis of patients from identification to testing to treatment initiation must be routinely carried out and qualitative methods should be also considered. An improved mechanism for tracking referrals from presumptive diagnosis to treatment commencement should be developed.

The MDR-TB diagnosis and treatment pathway is costly and long, even in settings where health care and diagnostic tests are provided free of charge at the point of delivery. Reducing costs and time for patients might improve retention in care [4]. In Bangladesh, despite reimbursement of transport costs for MDR-TB testing to eligible patients, the actual number of patients enrolled in care remains low. More support, including financial incentives, may be considered, especially for the poorest patients whose initial out-of-pocket expenditures may be a potential barrier of accessing health services. Finally, training of all health workers, especially at the peripheral level, and strong supervision and linkages need to be established throughout the diagnosis and treatment pathway.

Further studies are recommended to assess implementation of systems-wide interventions to improve identification, tracking, and ultimately treatment of MDR TB patients to minimize further infections.

In conclusion, low pre-diagnosis and pre-treatment attrition was found among MDR-TB patients under programmatic conditions in a large area of Bangladesh. However, several operational gaps were identified; especially low overall MDR-TB case detection, and several weaknesses in the existing reporting and recording system which highlight the urgent need for action to prevent MDRTB associated morbidity and mortality and community transmission of resistant TB strains.
